# High voltage-gain full-bridge cascaded dc-dc converter for photovoltaic application

**DOI:** 10.1371/journal.pone.0206691

**Published:** 2018-11-30

**Authors:** M. Zakir Hossain, Jeyraj A / L Selvaraj, N. A. Rahim

**Affiliations:** 1 UM Power Energy Dedicated Advanced Centre (UMPEDAC), University of Malaya, Kuala Lumpur, Malaysia; 2 Institute of Graduate Studies, University of Malaya, Kuala Lumpur, Malaysia; 3 Distinguish Adjunct Professor, Renewable Energy Research Group, King Abdulaziz University, Jeddah, Saudi Arabia; University of Science and Technology Beijing, CHINA

## Abstract

Over the past few years, high step-up dc-dc converters have been drawn substantial attention because of their wide-ranging application not only in the renewable energy sector but also in many other applications. To acquire a high voltage gain in photovoltaic (PV) and other renewable energy applications, a high step-up dc-dc converter is proposed in this paper. The proposed converter structure consists of a full-bridge (FB) module along with an input boost inductor and a voltage multiplier based on the Cockcroft-Walton (CW) principle with a parallel inductor. The key features of the proposed converter are: 1) high voltage gain with lower voltage stress on the switches, diodes and other passive elements without affecting the number of cascaded stages, 2) a minimum size of boost inductance and cascaded stage capacitance that ensures its compactness and low cost, and 3) a minimal number of major components. Circuit operation, steady-state analysis and various design parameters of the proposed converter are explained in details. In order to prove the performance of the theoretical analysis, a laboratory prototype is also implemented. The peak voltage gain and the maximum efficiency obtained are 11.9 and 94.6% respectively with very low input current ripple and output voltage ripple generated.

## Introduction

Solar resources are inexhaustible and their harvest and applications are environmental friendly. Power generation from solar energy through photovoltaic cells is recognized as one of the most susceptible technologies in renewable energy [1, 2]. However, poor efficiency of the energy conversion system is the major obstacle to their growth. Moreover, without additional instrumentation, the PV modules output voltage is relatively low and fluctuates with respect to sunlight intensity [3–5]. Hence, high gain high-efficiency dc-dc converter is essential to boost-up the PV output compatible with the required input voltage for different dc and ac loads. In addition, to enhance the energy supply accessibility of the poorly grid, battery and bidirectional converter can be utilized as a backup. The dc-dc converter having a higher gain along with high-efficiency characteristics are suitable during the battery discharging period in case of standard 48-V battery [4]. The hybrid distribution power system (using of fuel cell and/or ultracapacitor) and fuel cell electric vehicles (FCEV) also employs highstep-up converter because of their very low output voltage [6–8]. In addition, the vehicle to grid (V2G) technology implemented in the plug-in-hybrid electric vehicle (PHEV) requires highstep-up converter [9, 10]. Therefore, high gain, cost-effective dc-dc converters with the high-efficiency property are essential in renewable energy as well as many other applications.

In theory, traditional single-switch single-phase boost converter can attain infinite voltage step-up ratio at unity duty cycle. Complexities arise in the case of extremely high duty cycle such as in case of switch turn-off period is large. Voltage stress in the active devices are equal to the converter output voltage and it gets increased with high voltage applications, thereby escalating the price of converter devices. Moreover, because of the high current ripples, conduction losses and turn-off current of the power devices are high during high voltage-gain operation. Switching losses are also high due to the lack of the soft switching operation. To reduce these losses in conventional boost converters, many soft-switching techniques have been proposed and majorities of these improvements have been implemented in the power factor correction (PFC) system [11, 12]. Converters designed based on conventional boost and Ćuk topologies also needs to operate in very high duty cycle to achieve a higher step-up ratios[13].

A good number of dc-dc converters have been investigated till now to attain high voltage step-up ratio by avoiding excessive duty cycle and using either a step-up high-frequency transformer or coupled inductors [14–18]. Transformer action can be achieved by the utilization of coupled inductors which boosts up the converter gain. Although the dc/dc converter proposed in [14] offers higher efficiency at lower step-up ratio and power, the utilization of single switch causes high voltage and current stress resulting indisposition of higher rating switching devices. A dc-dc non-isolated converter consisting of single-switch and coupled inductor was investigated in [17]. For low power applications, this architecture provides high voltage ratio, low active device voltage stress, low conduction losses and low input current ripple. Moreover, exclusion of transformer in reduces the size, weight and overall complexity of the converter, which in turn lower the price.

The dc-dc converter designed by the switched capacitors (SC) principle can operate at higher temperatures than their inductor based counterparts [19]. The voltage conversion ratio can also be increased to a higher value by utilizing SC network in dc-dc converters [20–22], in which the capacitor is considered as some other voltage source to attain a high voltage gain. In [20], an *n*-stage high voltage ratio SC dc-dc converter is presented, which offers high voltage gain and wide-range operation by cascading *n*-stage of SC cells. To obtain a high gain in high voltage systems, a resonant SC converter is proposed in [21]. The beneficial features of this topology are the reduced output capacitance utilization and the lower capacitor power rating along with the soft switching operation. In addition, its output capacitors charge and discharge periodically by 180° phase shift that diminishes the output voltage ripple without any extra arrangements. However, the requirements of more passive elements increase the overall converter complexity. Coupled inductor and SC can be jointly employed in the dc-dc converter to gain high voltage ratio [23–26]. However, a voltage spike is created on the main switch in this type of architectures due to the leakage inductance stored energy which deteriorates the conversion efficiency.

The dc-dc converters offering high gain designed by cascaded diode-capacitor or diode-inductor cell rather than the use of coupledinductor or step up transformer have also been proposed by many researchers that offer high voltage conversion ratio with simple and robust structure [27–30]. In addition, the control techniques used in the traditional dc-dc converter architectures can be simply employed to these topologies. However, as the number of cascaded stage increase the majority of these type of cascaded structures suffer from higher active and passive devices voltage stresses as the number of cascaded stage increase. Moreover, the single switch single phase topology restricts the power handling capability of these converters. The benefits of the widely used traditional CW network are high voltage gain, low capacitor and diode voltage stresses, reduced size and cost-effectiveness. For this reason, CW multiplier is quite popular in many high step-up dc fields. A four-switch cascaded dc-dc converter utilizing CW multiplier cell has been proposed in [30] that provides high gain without employing line- or high-frequency transformer. Moreover, the voltage stresses of switch, diodes, and capacitors are lower, which is also independent of the number of cascaded stages. However, the high losses, i.e., the lower efficiency and higher boost inductor size make this topology unpopular.

In this paper, a high voltage gain full-bridge (FB) cascaded dc-dc converter has been proposed. In order to reduce the converter size and weight, the boost inductance size is reduced. To further enhance the voltage gain an extra inductor is inserted at the CW terminal, which facilitates a higher voltage conversion ratio than the conventional CW multiplier based converter. Although the use of four switches needs an extra isolated driver circuit, the proposed topology possess several adjuvant features: 1) lower voltage stresses of the active devices facilitate the use of low resistance, *R*_*DS(ON)*_ switch and Schottky diode cause the reduced losses, leading to the higher efficiency; 2) the use of the boost inductance and CW capacitance are reduced significantly resulting the prominent dynamic performances and compact converter size and weight; 3) high voltage gain can be attained and thus suitable for medium voltage or high voltage PV and many other energy applications. In addition, the proposed converter provideslower ripples in the input current as well as in output voltage.

The rest of the paper is planned as follows: Section 2 narrates the proposed converter operating principle in different operating modes followed by the steady-state analysis along with the design considerations of different parameters in Section 3. Section 4 presents the simulation and experimental validation of the proposed topology. The feasibility study of the designed converter is described in Section 5. Finally, the summary of this research is recounted as a conclusion in Section 6.

## Proposed converter operating principle

**Fig 1:**
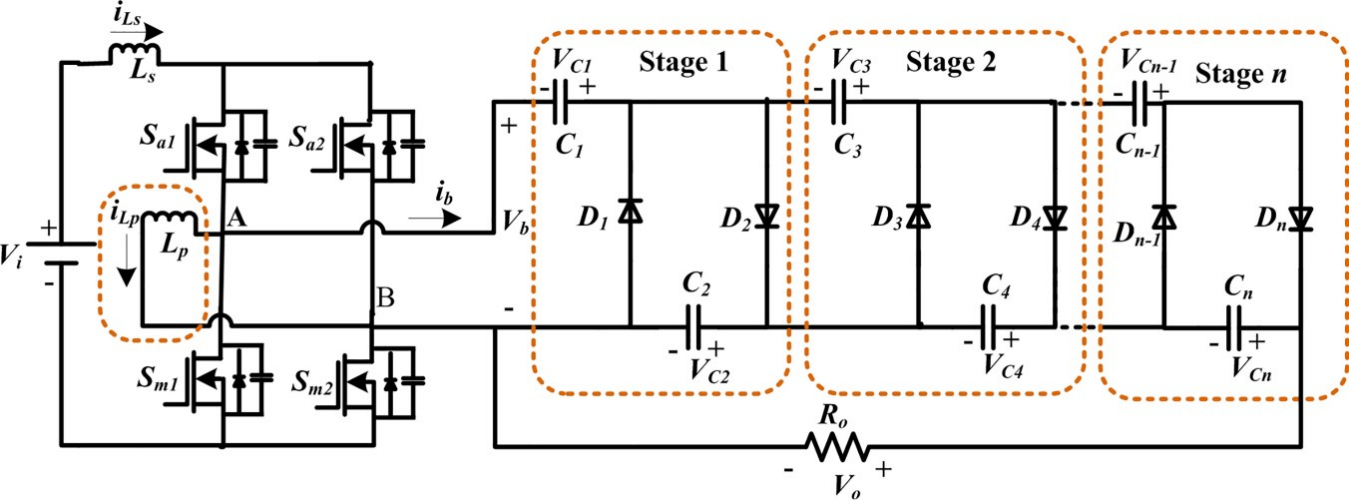
Proposed FB cascaded CW multiplier based dc-dc converter.

The Fig 1 shows the proposed converter circuit structure, which can be fed from a low voltage dc input source like dc power supply, battery, PV panel or even fuel cell. The suggested converter comprises an FB module (four switches are denoted as *S*_*a1*_, *S*_*a2*_, *S*_*m1*_, and *S*_*m2*_), one boost inductor (*L*_*s*_), one parallel inductor (*L*_*p*_) in order to further enhance the voltage gain, and an *n*-stage cascaded CW network as a voltage multiplier. Each stage of CW multiplier contains a pair of diodes and a pair of capacitors, hence the number of stage *n* = *N/2*, i. e., *N* = *2n* diodes and the equal quantity of capacitors exist in each stage.

Switches *S*_*a1*_, *S*_*m1*,_and *S*_*a2*_, *S*_*m2*_ operate in a complementary mode. For the analytical simplicity, the operating frequencies of the switches *S*_*a1*_ (*S*_*a2*_) and *S*_*m1*_ (*S*_*m2*_) are denoted as *f*_*sa*_ and *f*_*sm*_ respectively. In theory, the switching frequencies have to be selected as higher as possible to keep the capacitance and inductance as lower values as possible. In this work, switching frequency *f*_*sa*_ is kept much lower than *f*_*sm*_, and to regulate the required output voltage, *V*_*o*_ the duty ratio of *f*_*sm*_ is controlled, whereas the ripple of *V*_*o*_ changes with the varying *f*_*sa*_.

In order to simplify the operation principle and mathematical analysis of the developed converter, some assumptions have been made as follows:

1. All the capacitors used in this topology are large enough, thus, all capacitors voltages are identical, except the voltage of the first one, which is one-half of the other capacitors.
2. All circuit components such as active devices (switches and diodes) and passive devices (capacitors and inductors) are ideal, thus the losses and ripples are ignored.
3. The steady-state condition and continuous conduction mode (CCM) operation are considered.
4. During the inductor stored energy transfers to the CW multiplier, only one diode of the CW circuit is in conduction.

**Fig 2:**
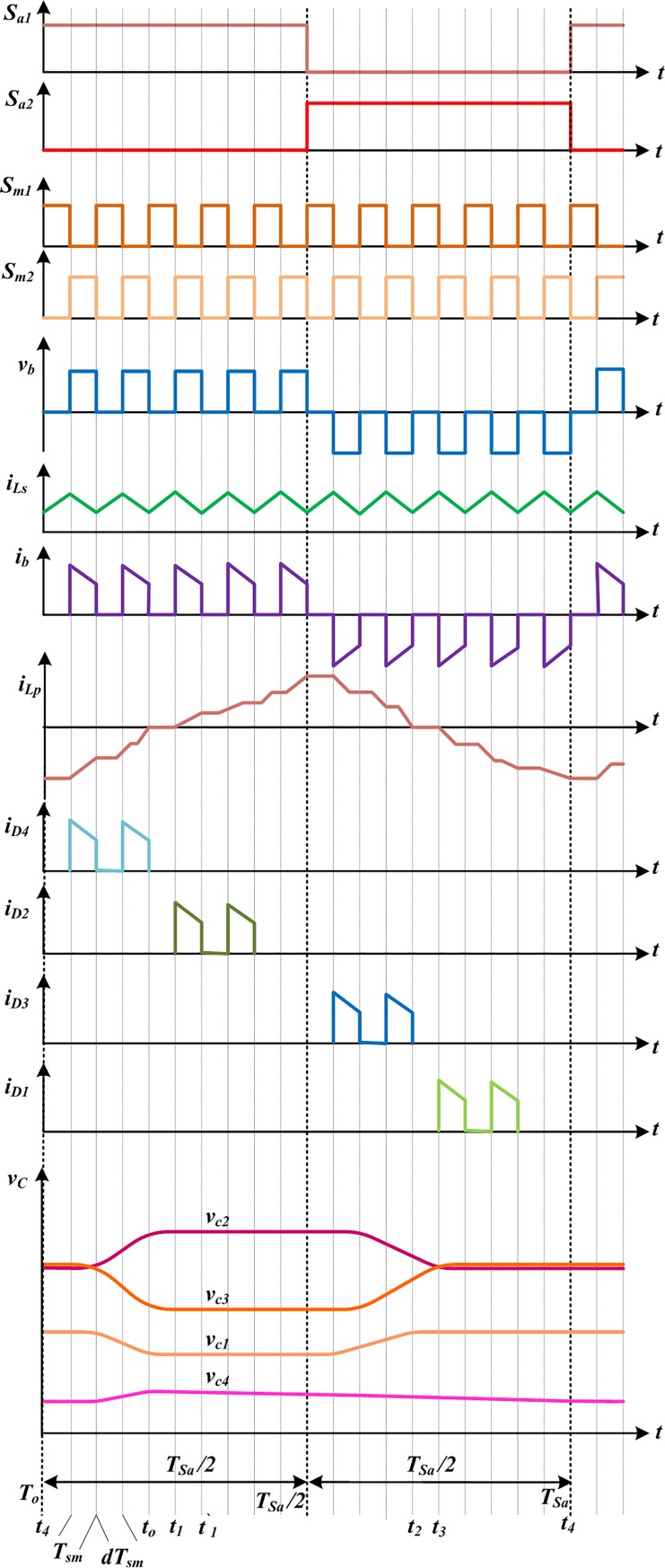
Ideal waveforms of the developed dc-dc converter during one switching period in CCM mode.

**Fig 3:**
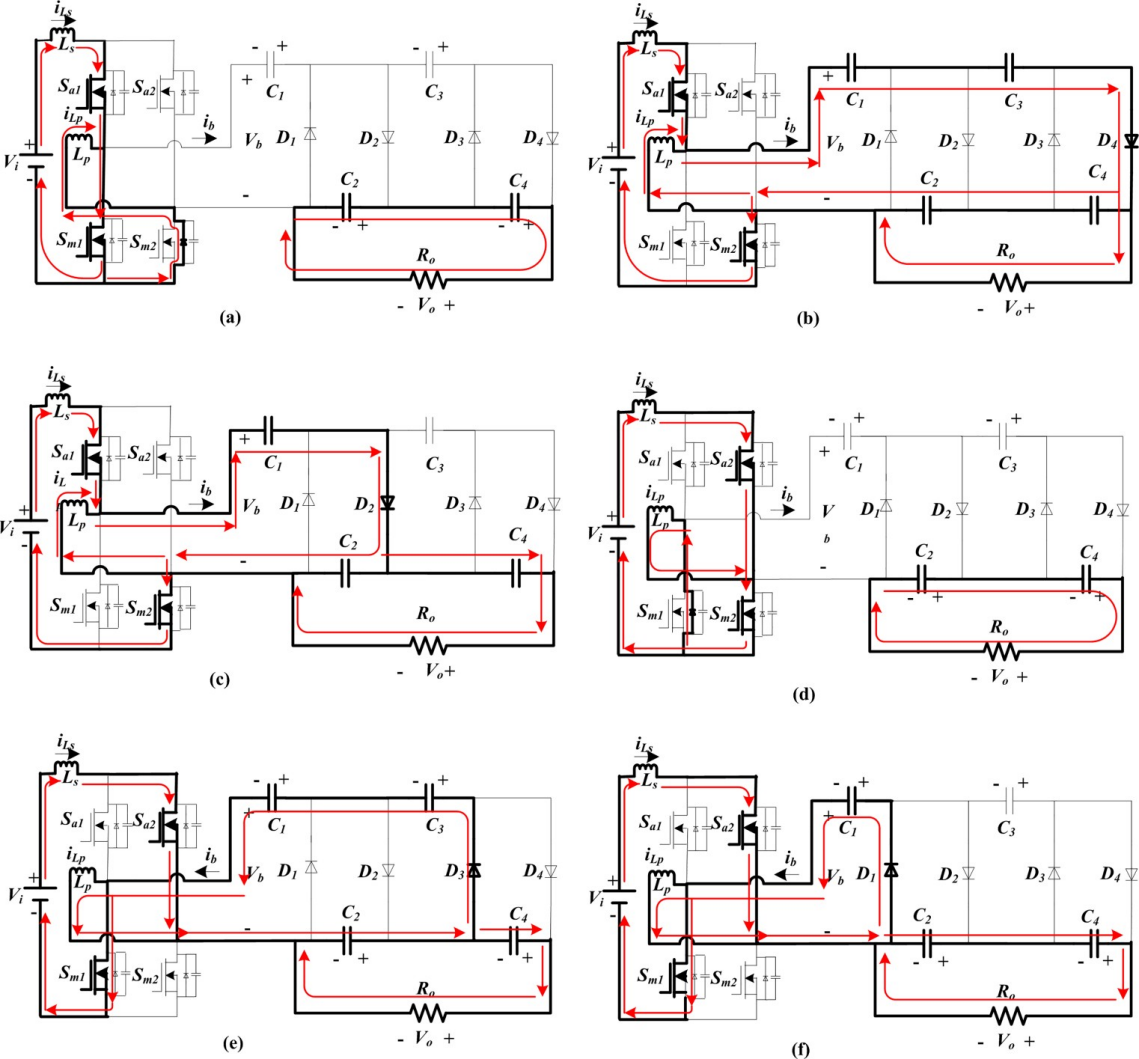
Operating stages of the proposed converter.

The ideal waveforms of the proposed topology for 2-stage CW multiplier are shown in Fig 2 for one switching period. It contains switching signals, bridge voltage and current, (*v*_*b*_ and *i*_*b*_), inductors currents, and diodes currents and voltages. Moreover, the current-flow paths of the developed converter for each operating stages are illustrated in Fig 3. As the alternating nature of *i*_*b*_, the CCM operating modes of the suggested topology can be broken up into two sections: during the positive interval and negative interval, and their time durations are [*T*_*o*_, *T*_*sa*_*/2*] and [*T*_*sa*_*/2*, *T*_*sa*_] respectively. In the first half-cycle (positive), only one even diode is in conduction with the order *D*_*4*_-*D*_*2*_, while in the negative half-cycle, only one odd diode conducts with the order *D*_*3*_-*D*_*1*_. In addition, during this first half-cycle, there are three operating stages displayed in Fig 3(A)–3(C), indicated as I, II-a and II-b. Likewise in the opposite interval, there are also three stages shown in Fig 3(D)–3(F), indicated as III, IV-a and IV-b. The circuit operation principles according to the operating stages in Fig 3 are explained in details as below.

1. Stage I [Fig 3(A)]: In this stage, switch *S*_*a1*_ and *S*_*m1*_ are switched ON, while the other two switches(*S*_*a2*_,*S*_*m2*_) and all the CW network diodes are switched OFF. The dc voltage source, *V*_*i*_ charges the boost and parallel inductors through the conducting switches *S*_*a1*_ and *S*_*m1*_, and the bypass diode of *S*_*m2*_ respectively. The capacitors in the bottom side,*C*_*4*_ and *C*_*2*_ (Fig 3(A)) supply current to the output, whereas the upper side capacitors *C*_*3*_ and *C*_*1*_ (Fig 3(A)) remain in floating.
2. Stage II [Fig 3(B) and 3(C)]: In stage II, *S*_*a1*_ and *S*_*m2*_ are switched ON, while *S*_*a2*_ and *S*_*m1*_ are switched OFF. The inductors and the dc input voltage source supply energy to the cascaded network by conducting various even group diodes. In stage II-a, as shown in Fig 3(B), diode *D*_*4*_ conducts, therefore, the bridge current, *i*_*b*_ charges *C*_*2*_ and *C*_*4*_, and discharges the *C*_*1*_ and *C*_*3*_ as well. In the next stage II-b, diode *D*_*2*_ conducts, thus, the bridge current, *i*_*b*_ charges the capacitor *C*_*2*_ and discharges *C*_*1*_;*C*_*4*_ supplies to the load, while *C*_*3*_ is floating as seen in Fig 3(C).
3. Stage III [Fig 3(D)]: In this stage, switch *S*_*a2*_ and *S*_*m2*_ are switched ON, and the opposite two switches (*S*_*a1*_ and *S*_*m1*_) in the FB and all CW network diodes are switched OFF. The dc source charges the input boost inductor, *L*_*s*,_ and the parallel inductor *L*_*p*_ through the conducting switches *S*_*a2*_ and *S*_*m2*_, and the bypass diode of *S*_*m1*_ respectively. Similar to stage I, the bottom side capacitors transfer energy to the load, and *C*_*3*_ and *C*_*1*_ remain in floating.
4. Stage IV [Fig 3(E) and 3(F)]: *S*_*a2*_ and *S*_*m1*_ are switched ON, while *S*_*a1*_ and *S*_*m2*_ are switched OFF. The cascaded voltage multiplier receives energy from the inductors and dc voltage source, *V*_*i*_ by conducting several odd group diodes. In stage IV-a, as shown in Fig 3(E), diode *D*_*3*_ conducts, thus, the bridge current discharges the capacitor *C*_*2*_ and charges the capacitors, *C*_*3*_ and *C*_*1*_, and *C*_*4*_ supplies to the load. In the next stage IV-b, diode *D*_*1*_ conducts, therefore, the capacitor *C*_*1*_ is charged by *i*_*b*_, while capacitors *C*_*4*_ and *C*_*2*_provide the load current and *C*_*3*_ is floating, presented in Fig 3(F).

## Proposed converter analysis and design

### Capacitor voltage

To extend the applicability of the developed converter exposed in Fig 1, the mathematical analysis is done for *n*-stage cascaded voltage multiplier. According to the aforementioned assumptions, the voltage across each capacitor of the cascaded stage can be expressed as:

vcj={Vc2,forj=1Vc,forj=2,3…,N (1)

where *v*_*cj*_ is the *j*th capacitor and *v*_*c*_ is the voltage in steady-state of all capacitors except the first one. From Fig 1, it is obvious that the output voltage, *V*_*o*_ is the same as the summation of all even capacitors voltage and can be written as:

$$V_{o}={nV}_{c}$$ (2)

Combining (1) and (2), the voltag eof each capacitor in the cascaded network for *n*-stage can be presented as:

vcj={Vo2n,forj=1Von,forj=2,3…N (3)

### Voltage gain expression and inductor current

In the stages I and III as presented in Fig 3, the voltage across the CW network, *v*_*b*_ = 0. Therefore, during the interval *t*_*o*_<*t*<*t*_*1*_, the boost input inductor (*L*_*s*_) current can be expressed as:

$$i_{Ls}(t_{1}-t_{o})=\frac{{V_{i}-v}_{Lp}}{L_{s}}(t_{1}-t_{o})$$ (4)

where *V*_*i*_ is the converter input dc voltage and *v*_*Lp*_ is the parallel inductor voltage. On the other hand, during the interval ${t_{1}<t<t}_{1}^{\prime }$, in the operating stages II and IV, the voltage across the CW multiplier, *v*_*b*_ = *V*_*o*_/2*n*, hence the boost inductor current during this period is:

$$i_{Ls}(t_{1}^{\prime }-t_{1})=\frac{V_{i}-v_{b}}{L_{s}}(t_{1}^{\prime }-t_{1})$$

=Vi−V02nLs(t1′−t1) (5)

According to the ideal wave shapes in Fig 2, current (*i*_*Lp*_) flows through the parallel inductor (*L*_*p*_) in different six operating stages as displayed in Fig 3. It is seen from Fig 2 that the ideal frequency of the current, *i*_*Lp*_ is identical to the switching frequency (*f*_*sa*_) of the switches *S*_*a1*_ and *S*_*a2*_. The time interval of *i*_*Lp*_ starts from *t*_*o*_ to*t*_*2*_ (through *t*_*1*_) as the first half cycle,and*t*_*2*_to *t*_*o*_ (through *t*_*3*_ and *t*_*4*_) is considered as the second half cycle. In stage I [Fig 3(A)], current flows through the parallel inductor during the interval *t*_*o*_<*t*<*t*_*1*_, can be expressed as:

$$i_{Lp}(t_{1}-t_{o})=\frac{1}{L_{p}}\int_{t_{0}}^{t_{1}}v_{Lp}dt$$ (6)

In stage II [(stages II-a and -b) in Fig 3(B) and 3(C)], during the interval *t*_*1*_<*t*<*t*_*2*_, the inductor (*L*_*p*_) current can be written as:

$$i_{Lp}(t_{2}-t_{1})=\frac{1}{L_{p}}\int_{t_{1}}^{t_{2}}v_{Lp}dt$$ (7)

$$i_{Lp}(t_{2}-t_{1})=\frac{1}{L_{p}}\int_{t_{1}}^{t_{2}}({V_{i}-v}_{Ls})dt$$

$$i_{Lp}(t_{2}-t_{1})=\frac{\left({V_{i}-v}_{Ls}\right)}{L_{p}}(t_{2}-t_{1})$$ (8)

where *v*_*Ls*_ is the voltage across the boost inductor during the period of *t*_*1*_<*t*<*t*_*2*_. Like stage I, in stage III [Fig 3(D)], during the time period*t*_*2*_<*t*<*t*_*3*_, the parallel inductor (*L*_*p*_) current can be expressed as:

$$i_{Lp}(t_{3}-t_{2})=\frac{1}{L_{p}}\int_{t_{2}}^{t_{3}}v_{Lp}dt$$ (9)

Similar as stage II, in stage IV [(stages IV-a and-b) Fig 3(E) and 3(F)], the parallel inductor (*L*_*p*_) current during the interval *t*_*3*_<*t*<*t*_*0*_, can be determined as:

$$i_{Lp}(t_{o}-t_{3})=\frac{1}{L_{p}}\int_{t_{3}}^{t_{o}}v_{Lp}dt$$ (10)

$$i_{Lp}(t_{o}-t_{3})=\frac{1}{L_{p}}\int_{t_{3}}^{t_{o}}(v_{Ls}-V_{i})dt$$

$$i_{Lp}(t_{o}-t_{3})=\frac{\left(v_{Ls}-V_{i}\right)}{L_{p}}(t_{o}-t_{3})$$ (11)

From Fig 2, it is clearly seen that the time intervals(*t*_*o*_-*t*_*1*_) and (*t*_*1*_-*t`*_*1*_) are exactly equal to the intervals *dT*_*sm*_ and (1-*d)T*_*sm*_, where *d* is the duty ratio and *T*_*sm*_ (1/*f*_*sm*_) is the time period of the switching signal of switches *S*_*m1*_ and *S*_*m2*_.

Therefore, submitting *dT*_*sm*_ and (1-*d)T*_*sm*_ in the place of time in (4) and (5), and then employing the volt-second balance principle to the boost inductor (*L*_*s*_), the voltage gain of the designed converter can be expressed as:

$$M_{V}=\frac{V_{0}}{V_{i}}=\frac{1+d}{1-d}2n$$ (12)

**Fig 4:**
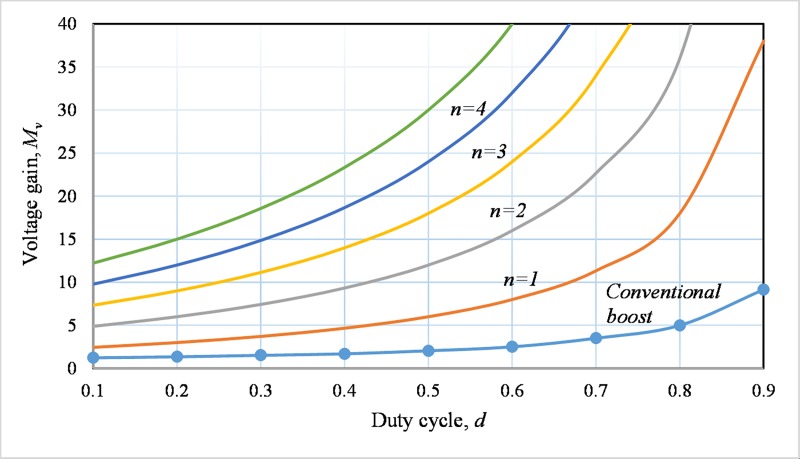
Graphical representation of gain changes due to the change in the duty cycle for the suggested converter for*n* = 1to 5 and the conventional boost dc-dc converter.

The relationship between the voltage step-up ratio and duty cycle of the proposed converter is shown in Fig 4 for *n* = 1 to 5 and compared with the performance of the traditional boost converter. It is clearly seen from Fig 4 that the voltage gain is higher than the boost converter by avoiding the operation in very high duty cycle. For comparison, the voltage gains of different high gain dc-dc conversion topologies are summarized in the first row of Table 1.

**Table 1:** Comparison of different parameters of the developed dc-dc cascaded converter with others.

Parameter	Proposed converter	Converter in [30]	Converter in [29]	Converter in [28]	Converter in [34]
Topology	Fig 1	Fig 7	Fig 8	Fig 4	Fig 7
**Voltage Gain**	*{(*1*+d)/(*1*-d)}*2*n*	2*n/(*1*-d)*	*(n+d)/(*1*-d)*, *n* is odd	(*n*+1)/(1-*d*)	2*n*/(1-*d*)
			*(n+*1-*d)/(*1*-d)*, *n* is even		
**Number of major components**	6+4*n*	5+4*n*	4+4*n*	4+4*n*	10+4*n*
**Voltage stress on switch**	*V*_*i*_(1+*d*)/(1-*d*)	*V*_*i*_/(1-*d*)	*V*_*i*_/(1-*d*)	*V*_*i*_/(1-*d*)	*V*_*i*_/2(1-*d*)
**Voltage stress on diode**	2*V*_*i*_(1+*d*)/(1-*d*)	2*V*_*i*_/(1-*d*)	*V*_*i*_/(1-*d*)	*V*_*i*_/(1-*d*)	2*V*_*i*_/(1-*d*)
**Voltage stress on capacitor**	*V*_*Cj =*_ *V*_*i*_(1+*d*)/(1-*d*) for *j* = 1	*V*_*Cj =*_ *V*_*i*_/(1-*d*) for *j* = 1	*V*_*Cj1*_ = *V*_*Cj2*_ = *jV*_*i*_/(1-*d*) for *j* = 1, . . . . . ., *N*	*V*_*Cj1*_ = *jV*_*i*_*/(*1-*d*) For *j* = 1, . . ., *N*	*kV*_*i*_/2(1-*d*)
	*V*_*Cj =*_ 2*V*_*i*_(1+*d*)/(1-*d*) for *j* = 2, . . . . . . ., *N*	*V*_*Cj =*_ 2*V*_*i*_/(1-*d*) for *j* = 2, . . . ., *N*		*V*_*Cj2*_ = *V*_*i*_*/(*1-*d*)	

N.B.: *k* is the turns ratio of transformer.

### Design example

In this subsection, the maximum stresses regarding voltage and current on the different major components of the suggested converter have been discussed. In addition, to optimize the parameters design, the values of the passive components has explained based on their stresses and acceptable ripples of the input current and output voltage.

#### Inductor sizing

**Fig 5:**
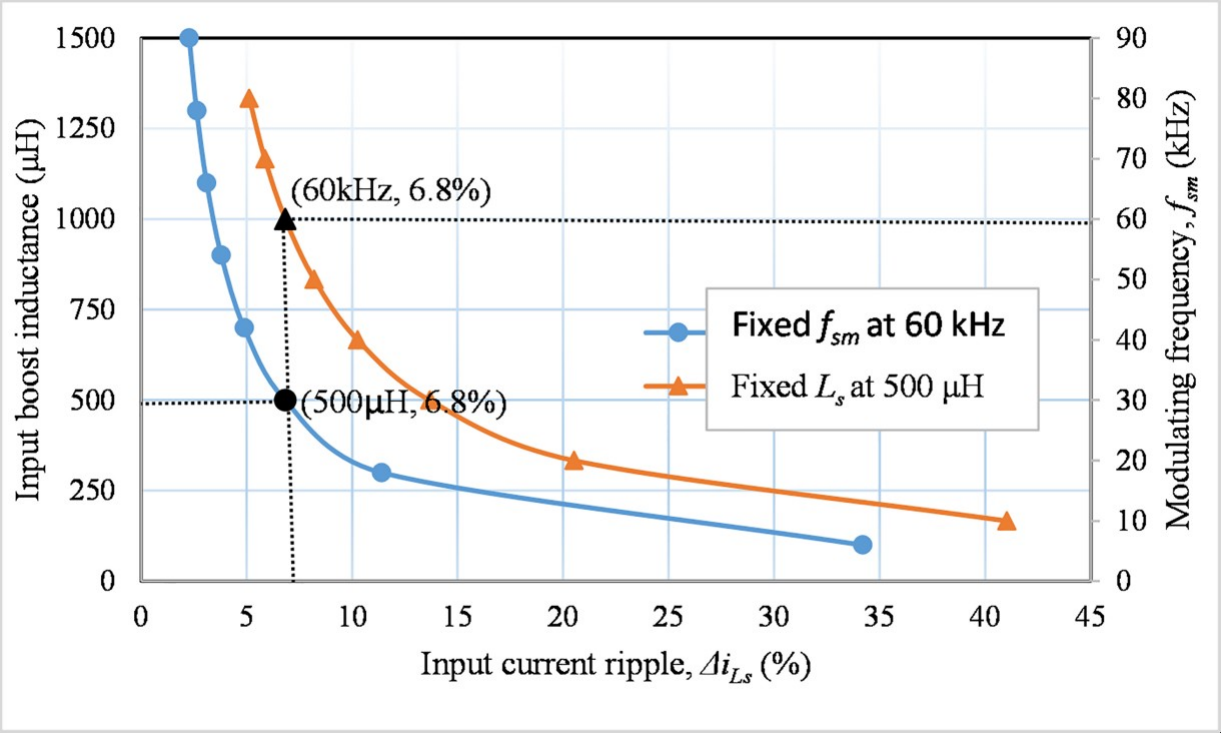
Input current ripple as a function of input boost inductance.

The boost inductor is an important design parameter which determines the input current ripple of the proposed converter. As mentioned earlier, the proposed converter has been designed in such a way that it is suitable for a nonlinear source like PV system. However, ripple current remarkably deteriorates PV system efficiency significantly. Considering the size and cost of the inductor, it should be chosen in such a manner that ripple remains to its minimum. Boost inductance, *L*_*s*_ can be determined by:

$$L_{s}=\frac{V_{i}.d}{I_{Ls.pk.}f_{sm.}\Delta i_{Ls}.pk}$$ (13)

where *I*_*Ls*.*pk*_ is the maximum input current and Δ*i*_*Ls*.*pk*_ is the percentage of input current ripple. For a fixed input voltage and duty cycle, from (13) it is seen that the input current ripple depends on the input boost inductance and the switching frequency (*f*_*sm*_) of the lower two switches (*S*_*m1*_ and *S*_*m2*_) in Fig 1. The relationships among these variables are presented in Fig 5.Thesolid line of Fig 5 represents the boost inductance versus current ripple at the constant switching frequency, *f*_*sm*_ is 60 kHz. In addition, the input ripple versus switching frequency is presented by the dashed line at constant, *L*_*s*_ = 500 μH. For both of the cases, the current ripple should be same and is 6.8%. Hence, for switching frequency *f*_*sm*_ is 60 kHz and Δ*i*_*Ls*.*pk*_ is 6.8%, the boost inductance is chosen as 500 μH for this topology. The maximum energy stored in the boost inductor *L*_*s*_ can be determined as

$$W_{Ls}=\frac{1}{2}L_{s}.I_{Ls.pk}^{2}=\frac{1}{2}L_{s}{.(\frac{V_{i}.d}{L_{s}.f_{sm.}\Delta i_{Ls}.pk})}^{2}$$ (14)

In the case of parallel inductance, the value can also be calculated as:

$$L_{p}=\frac{V_{b}.d}{I_{Lp.pk.}f_{sm.}\Delta i_{Lp}.pk}$$ (15)

where *I*_*Lp*.*pk*_ is the parallel inductor (*L*_*p*_) maximum current and Δ*i*_*Lp*.*pk*_ is the percentage of the ripple of the input current. The maximum energy stored in *L*_*p*_ can also be determined as

$$W_{Lp}=\frac{1}{2}L_{p}.I_{Lp.pk}^{2}=\frac{1}{2}L_{p}{.(\frac{V_{i}.d}{L_{p}.f_{sm.}\Delta i_{Lp}.pk})}^{2}$$ (16)

#### Capacitor sizing and voltage stress

**Fig 6:**
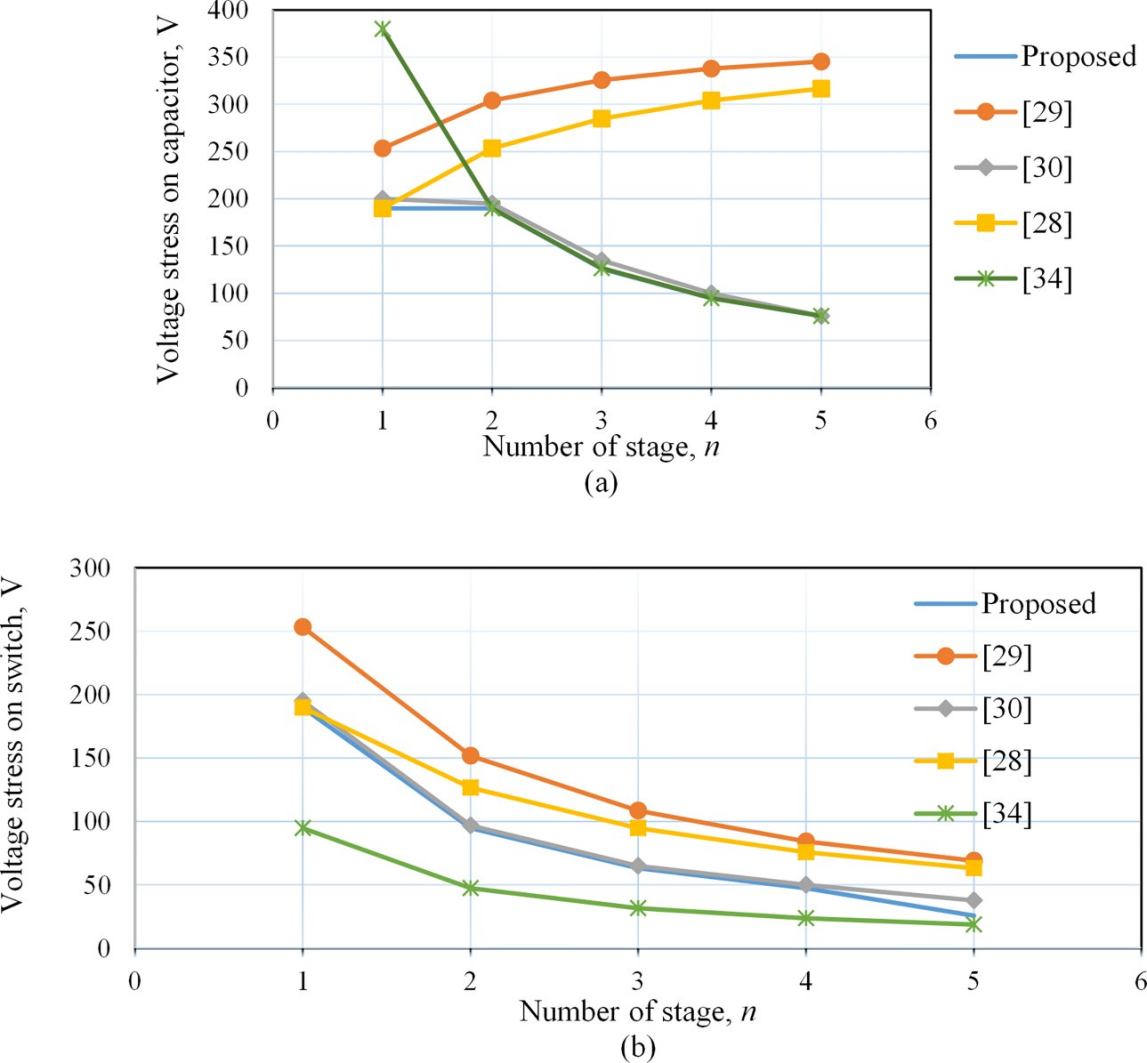
Voltage stresses on different devices for the proposed converter and others at duty cycle *d* = 0.5 and output voltage at *V*_*o*_ = 380V. Thus, the required input voltage decreases as the number of stage increase. (a) Capacitor voltage stress. (b) Switch voltage stress.

As mentioned above, all the capacitors (excluding the first one) possess very high capacitance with the same voltage level, while the voltage of the first capacitor is one-half of the others. Hence, from (3),the maximum voltage stress on the CW capacitors is *V*_*o*.*pk*_/*2n*, except the first one which is *V*_*o*.*pk*_/*n*, where *V*_*o*.*pk*_ is the peak output voltage. The voltage stresses on the individual capacitor of the developed and other converter topologies are listed in the fifth row of Table 1.Thecapacitor voltage stress for the proposed converter and converter reported in [30], depends only on the duty ratio and dc input voltage as mentioned in Table 1, whereas for the other converters it strongly depends on the number of cascaded stages (*n*). The voltage stresses on the different devices for the proposed converter and others are demonstrated in Fig 6 at a constant duty cycle, *d* = 0.5 anda constant output voltage,*V*_*o*_ = 380V. Thus, from the voltage gain expressions of these converters mentioned in Table 1, it is seen that the requirement of the input voltage (*V*_*i*_) decreases as the number of stage increase. For example, in the case of a constant, *d* = 0.5 and *V*_*o*_ = 380V, if the number of stages, *n* = 2, then*V*_*i*_ for the proposed converter is 31.67 V, while this value is 76V for the converter reported in[29]. Hence, the capacitor voltage stress is in present topology than the previous ones as shown in Fig 6(A).

From (3) and (12), the capacitor voltage with respect to duty cycle can be expressed by:

vcj={Vi(1+d)1−d,forj=12Vi(1+d)1−d,forj=2,3…,N (17)

From (17) it is seen that the individual capacitor voltage varies with the variation of the input voltage and duty ratio rather than the number of stages like the other topologies mentioned in the literature. Although theoretically all capacitor voltages are same during loaded condition the voltage drops and ripples of the capacitors cannot be ignored. According to the current-fed analysis[31], which is less complex than its counterpart voltage-fed analysis [32, 33], the voltage ripple of the individual capacitor is as follows:

$$\Delta V_{cj}=\frac{I_{o.av.}T_{sa}}{C}\left(\frac{N-j+1}{2}\right)\;for\;j=1,2,3,\ldots ,N$$ (18)

where *I*_*o*.*av*_ is the average output current and *T*_*sa*_ is the time period of the alternating frequency.

**Fig 7:**
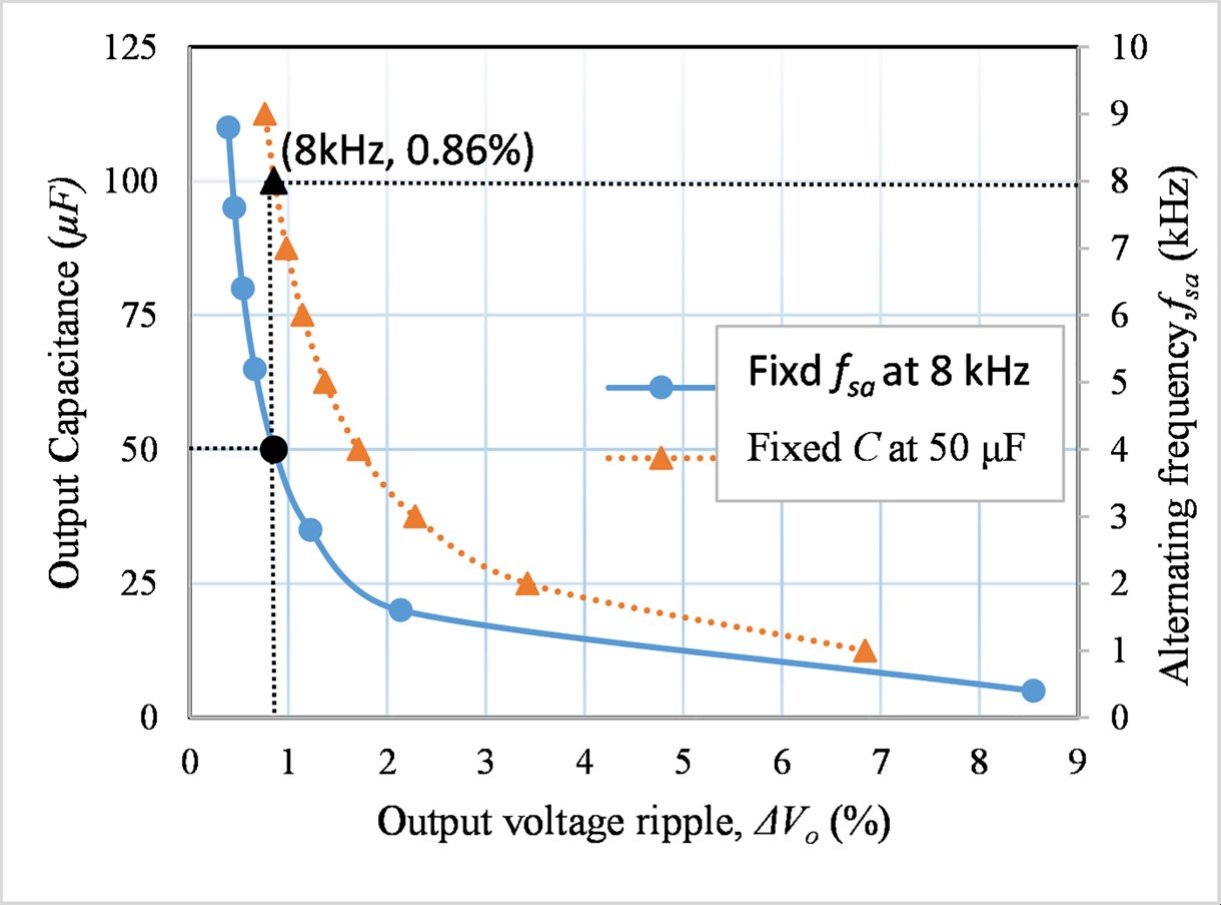
Output voltage ripple changes with the change in output capacitance.

For a fixed output current and number of cascaded stage, from (18) it is seen that the ripple in the output voltage depends on the bottom side capacitance at the output and the switching frequency, (*f*_*sm*_) of the upper two switches (*S*_*a1*_ and *S*_*a2*_) in Fig 1. The relationships among these variables are shown in Fig 7. The solid line in Fig 7 represents the capacitance versus voltage ripple at the constant switching frequency, *f*_*sa*_ is 8 kHz. Moreover, the switching frequency, *f*_*sa*_ versus ripple is presented by the dashed line at constant *C* = 50 μF. For both of the cases, the voltage ripple (*Δv*_*C*_) should be the same at 0.86%. Hence, for switching frequency *f*_*sa*_ of 8 kHz and *Δv*_*C*_ of 0.86%, the capacitance is taken as 50 μF for the proposed converter. The capacitor stored energy can be expressed as:

$$w_{cj}=\frac{1}{2}C_{j}v_{cj}^{2}$$ (19)

Submitting (17) and (18) into (19),

wcj=Io.av.Tsa2ΔVcj(N−j+12){(Vi(1+d)1−d)2,forj=1(2Vi(1+d)1−d)2,forj=2,3…,N (20)

#### Stresses of voltage and current on the switch

The rating and cost of the switching devices greatly depend on their current stress and voltage stress. The peak stresses due to voltage and current on the switch are *V*_*o*.*pk*_*/2n* and *I*_*Ls*.*pk*_ respectively, where *I*_*Ls*.*pk*_ is the maximum input current. The voltage stress on the switch can be written as:

$$v_{o.pk}=\frac{V_{o}}{2n}$$ (21)

The switch voltage stress with respect to theinput voltage and duty cycle can be found by combining (12) and (21).

$$v_{o.pk}=\frac{V_{i}(1+d)}{1-d}$$ (22)

The analysis of voltage stress on each switch is done in asimilar way to the capacitor as mentioned above, i.e., at *d* = 0.5 and *V*_*o*_ = 380V, as shown in Fig 6(B). Although the voltage stress of the switch for present converter is very close to that of the converters reported in [30, 34] shown in Fig 6(B), however, still it is lower than those reported for other converters [28, 29].

#### Stresses of voltage and current on diode

The diode peak voltage stress is twice the switching devices which is *V*_*o*.*pk*_*/n*, and maximum current stress is *I*_*b*.*pk*_, where *I*_*b*.*pk*_ is the peak input current of the CW multiplier.

**Fig 8:**
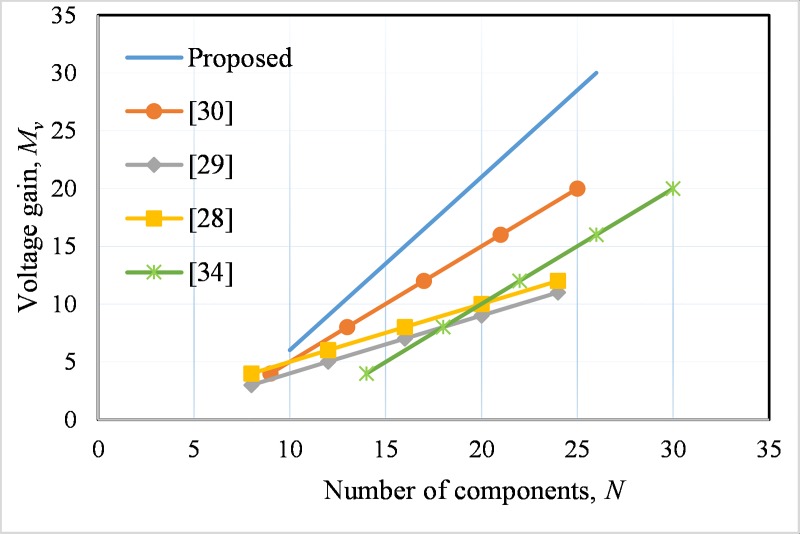
Voltage gain at different component numbersfor duty ratio,*d* = 0.5, *V*_*o*_ = 380V for the developed converter and other topologies.

The major components required in this type of dc-dc converter includes the passive devices such as inductor and capacitor and the active devices likeswitch (MOSFET/ Thyristor) and diode. For comparison, the number of components of the proposed topology and the othersis presented in the second row of Table 1. In addition, the number of major components versus the voltage gain is demonstrated in Fig 8. It is evident from Fig 8 that relatively less number of components is required for the proposedarchitecture than that of the other topologies to achieve the same voltage gain. For example, gain *M*_*v*_ = 12, the developed converter requires stage*n* = 2 and total major components are 14 at *d* = 0.5. While for the same number of stages and duty cycle converter in [30] provides voltage gain only 8 at atotal number of main components of 13. Likewise, converters in [29] and [28], the voltage step-up ratios are only 5 and 6 respectively at the total number of themain components requirement are 12 for each at similar values of *n* and *d*. However, the worst case is for the converter in [34], which needs 22 number of major components to achieve same voltage gain, 12.

The Table 2 represents the performance of some boost type dc-dc converters suitable for renewable energy (e.g. PV, FC, etc.) applications are compared with the suggested converter. The voltage gain is higher for the converter in [35]than the others, whereas the ON time of the switch(s) is kept lower for the proposed converter and the converter in [26]. In addition, although the measured efficiency is slightly higher of the converter in [22], however, its input current ripple much higher than the developed converter. Furthermore, converter in [26], offers least output voltage ripple, however, it suffers by poor efficiency at the rated power.

**Table 2:** Comparison between the developed converter and some other converters applicable in PV application.

Parameter	Converter in [22]	Converter in[35]	Converter in [26]	Proposed
Topology	Fig 6(A)	Fig 1	Fig 7	Fig 1
Output power, *P*_*o*_(W)	100	225	120	250
Duty cycle, *D*	0.65	0.65	0.5	0.5
Voltage gain, *M*_*v*_	8.44	11.11	9	8.44
Output voltage ripple, *ΔV*_*o*_(%)	1	<1	0.6	0.86
Input current ripple, *ΔI*_*i*_(%)	20	—	—	6.8
Efficiency, *η*(%)	94.86	93.2	90	94.5

## Experimental and simulation evaluations

A laboratory experimental setup of the proposed dc-dc converter has been implemented, the outcome of which validates the theoretical and simulation performance. The specifications of the designed converter and the description of the devices are disclosed in Tables 3 and 4 respectively. The simulation of the different parameters is executed in MATLAB/Simulink platform. The modeling of the proposed converter is performed for dc input voltage of 30~60 V with an output of 380 V, which is compatible with single phase 230 V (ac) inverter for PV application.

**Table 3:** Specifications of the proposed converter prototype.

Parameters	Value
Output voltage, *V*_*o*_	380 V
Input dc voltage, *V*_*i*_	30~60 V
Alternating frequency, *f*_*sa*_	8 kHz
Modulation frequency, *f*_*sm*_	60 kHz
No. of stage, *n*	2

**Table 4:** Components description of proposed converter prototype.

Components description and symbol	Value / Part no.
MOSFET, *S*_*a1*_, *S*_*a2*_, *S*_*m1*,_ *S*_*m2*_	C3M0120090D (SiC)
Diode, *D*_*1*_ *~ D*_*4*_	IDH10S120 (SiC)
Capacitor, *C*_*1*_ *~ C*_*4*_	4× 50 μF Film capacitor
Boost inductor, *L*_*s*_	500 μH
Parallel inductor, *L*_*p*_	100 μH
Voltage sensor	LEM LV 25-P
Gate driver IC	HCPL-3120
Controller, DSP	TMDSDOCK28335

**Fig 9:**
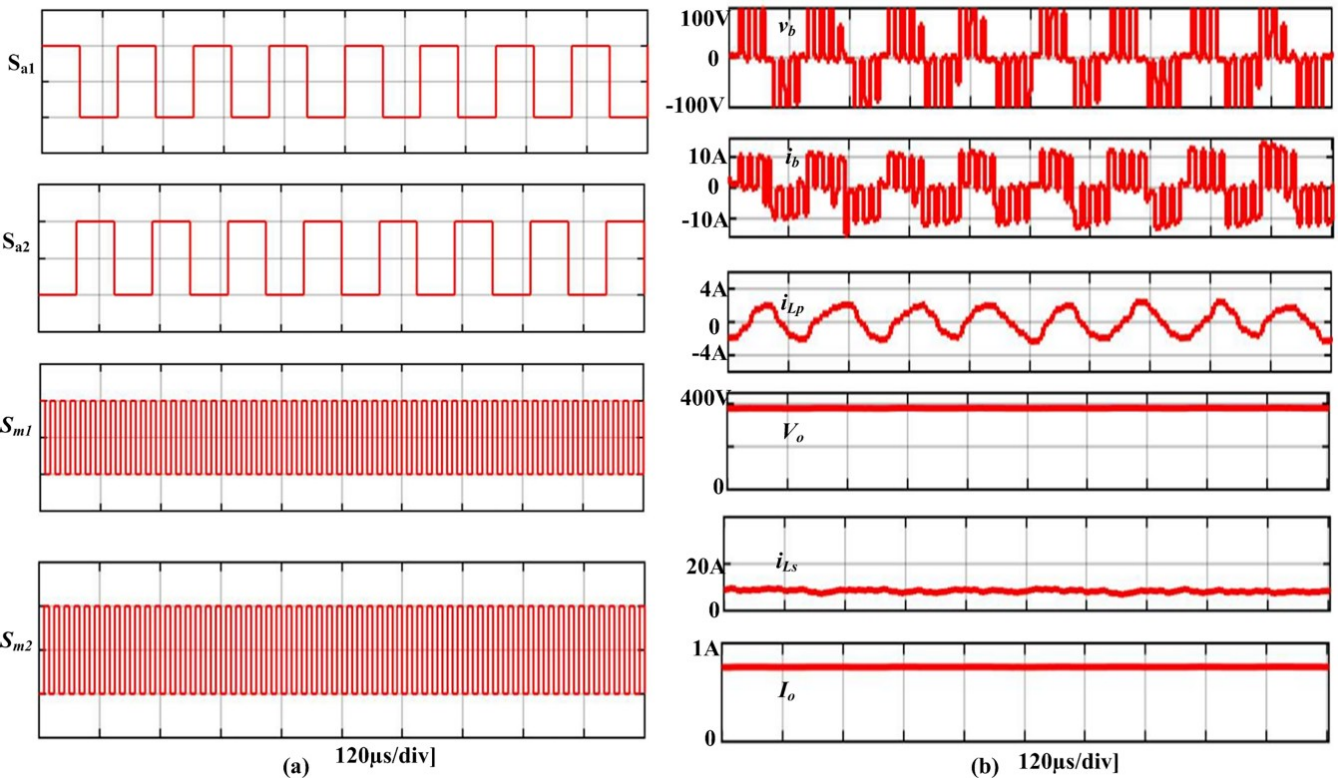
Simulation results of the developed FB cascaded CW converter.

**Fig 10:**
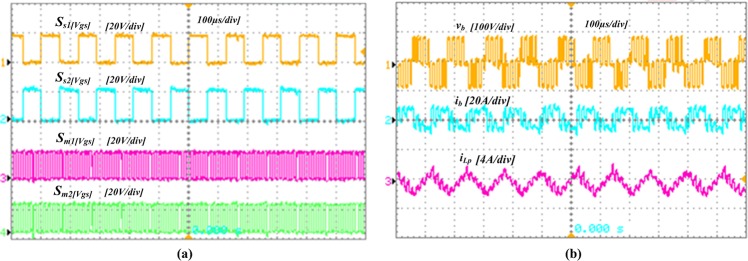
Experimental wave shapes of the developed converter. (a) Gate signals: *S*_*a1*_*(V*_*gs*_*)*, *S*_*a2*_*(V*_*gs*_*)*, *S*_*m1*_*(V*_*gs*_*)* and *S*_*m2*_*(V*_*gs*_*)*. (b) CW multiplier terminal (or FB) voltage and current, *v*_*b*_ and *i*_*b*_ respectively, and parallel inductor current, *i*_*Lp*_.

**Fig 11:**
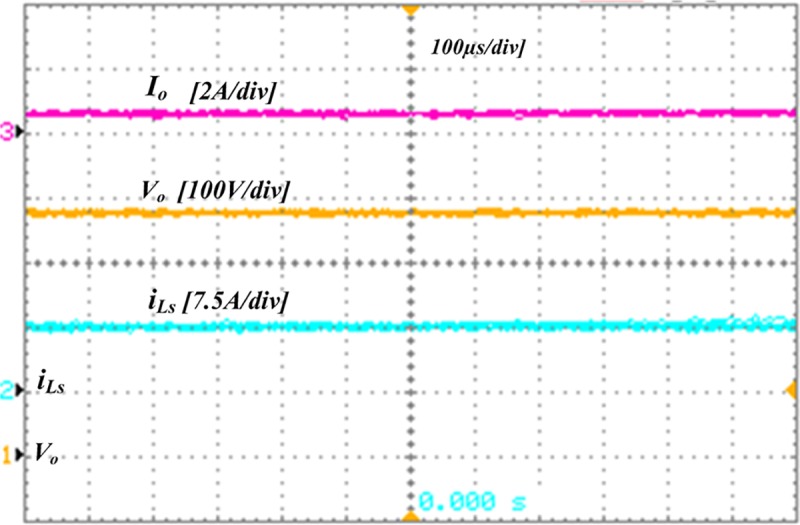
Experimental results. The output voltage, *V*_*o*_, boost inductor current, *i*_*Ls*_ and output current, *I*_*o*_.

The proposed converter can handle a maximum power of 1000 W. However, for convenience, all the simulation and experiments has been accomplished for 250 W load. Fig 9 demonstrates the simulation results of the developed converter architecture in the steady-state mode. Fig 9(A) presents the switching waveforms of the four MOSFETs *S*_*a1*_, *S*_*a2*_, *S*_*m1*,_and *S*_*m2*_, in which first two operate with an alternating frequency *f*_*sa*_, while the second two operate with *f*_*sm*_. Moreover, the simulation waveforms of FB terminal voltage and current, *v*_*b*_ and *i*_*b*_ respectively, the boost and parallel inductors current, *i*_*Ls*_ and *i*_*Lp*_ respectively, and output voltage, *V*_*o*_ and current, *I*_*o*_ are displayed in Fig 9(B). Fig 10 presents the experimental results, in which the gate signals are shown in Fig 10(A) and Fig 10(B) presents the *v*_*b*_, *i*_*b*,_ and *i*_*Lp*_. In addition, Fig 11 displays the experimental waveforms of *V*_*o*_, *I*_*o*,_ and *i*_*Ls*_. It is clearly seen from the Figs 9–11 that the experimental outcomes agree well with those of the simulation results.

**Fig 12:**
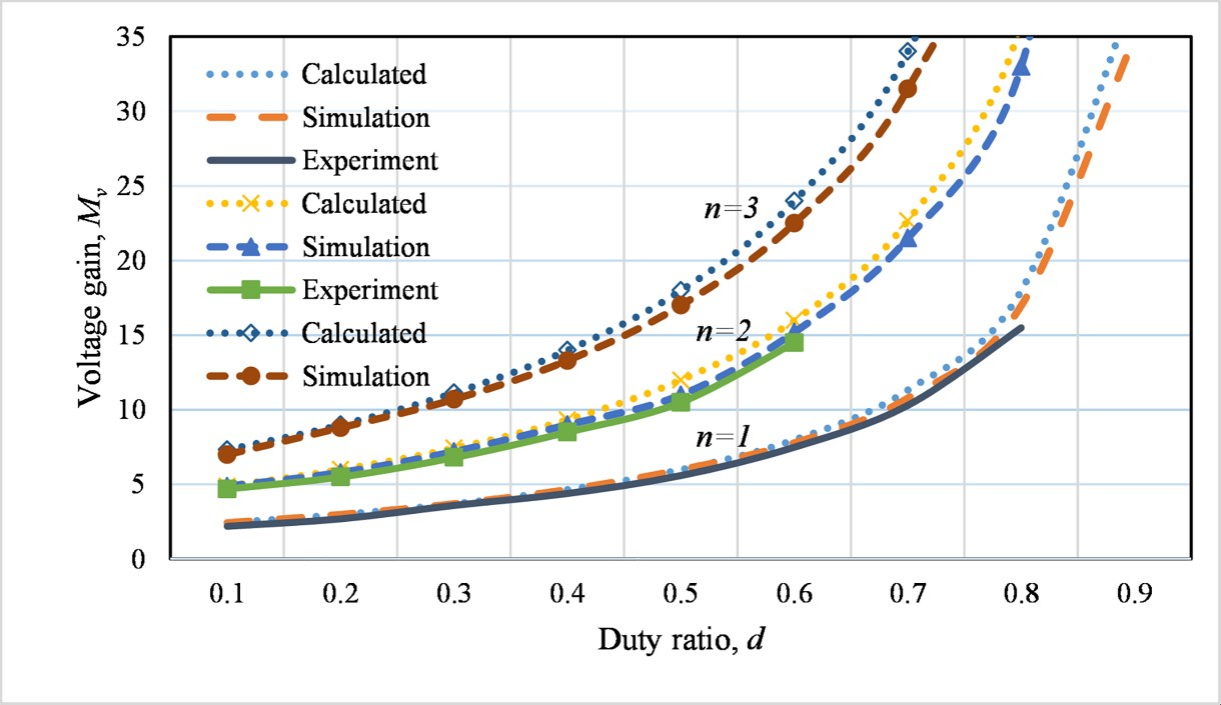
Calculated, simulation and experimental voltage gain, *M*_*v*_ versus duty ratio, *d* for the developed converter for *n* = 1 to 3 at 50% load.

The Fig 12 presents the calculated, simulation and the experimental voltage gain of the designed converter for various duty ratio. The calculated and simulation analysis is performed for the cascaded stage, *n* = 1, 2 and 3 for the duty cycle, *d* = 0 to 0.9, while the experiment is done for *n* = 1 and 2 cascaded stages with *d* = 0 to 0.8. The simulated and experimental voltage gains are well agreed up to a certain value of duty cycle. However, the simulated and experimental results slightly differ from the theoretical voltage gain. This is due to parasitic effects of various components when they are operating in high duty cycle.

The power loss distribution among the key components of the proposed converter are described in brief step-by-step in this sub-section. At first the power loss on the switching device as MOSFET can be expressed as:

$$P_{L(MOS)}=P_{SW(MOS)}+P_{CON(MOS)}$$ (23)

where *P*_*SW(MOS)*_ and *P*_*CON(MOS)*_ are the MOSFET switching and conduction losses respectively. The switching loss can be written as:

$$P_{SW(MOS)}=\frac{1}{2}.V_{DS}.I_{D}.t_{ON}.f_{SW}+\frac{1}{2}.V_{DS}.I_{D}.t_{OFF}.f_{SW}$$ (24)

where *V*_*DS*_, *I*_*D*_, *t*_*ON*_, *t*_*OFF*_ and *f*_*SW*_ are the MOSFET drain-source voltage, drain current, turn ON and OFF time and the switching frequency respectively. The switching frequency for MOSFETs *S*_*a1*_ and *S*_*a2*_is taken as 8 kHz, while for *S*_*m1*_ and *S*_*m2*_ is 60 kHz.

The conduction losses can be expressed as:

$$P_{CON(MOS)}={I_{RMS(MOS).}^{2}R}_{DS(ON)}$$ (25)

where $I_{RMS(MOS)}^{2}$ and *R*_*DS(ON)*_are the RMS current flows through the MOSFET and ON state resistance of the MOSFET respectively. Therefore, from the experimental data and data sheet of C3M0120090D MOSFET (used in this work), the total loss of a switch is:

$$P_{L(MOS)}=0.92+0.77=1.69\;W$$ (26)

Power loss on diode can be determined by multiplying the diode forward voltage drop, *V*_*F*_by the average current passes through diode, *I*_*d*_*(av)* during one switching cycle. Hence from data sheet of IDH10S120 diode and experimental average current, total diode losses:

$$P_{L(DIODE)}=V_{F}.I_{d(av)}=0.85\;W$$ (27)

The power dissipation on capacitor (B32776G4506K000 film capacitor used in this work) can be calculated as:

$$P_{L(CAP)}=I_{RMS(CAP).}^{2}{ESR}_{\left(CAP\right)}=0.65\;W$$ (28)

The inductor loss is the combination of core loss, *P*_*L(CORE)*_ and winding loss *P*_*L(WIND)*_. *P*_*L(CORE)*_ can be calculated by multiplying the effective volume of the core, *V*_*e*_ and the core loss per unit volume, *P*_*(C/V)*_ as:

$$P_{L(CORE)}={V_{e}.P}_{\left(C/V\right)}$$ (29)

Similarly the inductor winding loss can be expressed as:

$$P_{L(WIND)}=I_{L(AV)}^{2}.R_{DC}+I_{L(AC-RMS)}^{2}.R_{DC}=I_{L(AV)}^{2}.R_{DC}+\frac{I_{L(P-P)}^{2}}{value\;of\;AWG}.R_{DC}$$ (30)

where *I*_*L(AV)*_, *I*_*L(AC-RMS)*_, *I*_*L(P-P)*_ and *R*_*DC*_ are the inductor average current, inductor AC RMS current, inductor peak-peak ripple current magnitude and winding DC resistance respectively. The VISHAYIHV15BZ500 and BOURNS JW MILLER 1130-101K-RC devices are used in this work as input boost inductor and parallel inductor respectively. Thus, from the experimental results and inductor data sheet the total inductor losses is as:

$$P_{L(IND)}=P_{L(CORE)}+P_{L(WIND)}=2.52\;W$$ (31)

Therefore, the total calculated power loss of the developed converter is

$$P_{LOSS}={4*P}_{L(MOS)}+{4*P}_{L(DIODE)}+{{4*P}_{L(CAP)}+P}_{L(IND)}=15.30W$$ (32)

From the experiment it is observed that the measured power loss is 14 W, which is slightly lower than the above calculated power loss. This is because that the power loss is calculated for diode and capacitor by considering 25°C temperature. However, the junction temperature of these devices increases during power dissipation, which leads the decrease of forward voltage drop of diode and ESR of capacitor and resulting the less power loss.

**Fig 13:**
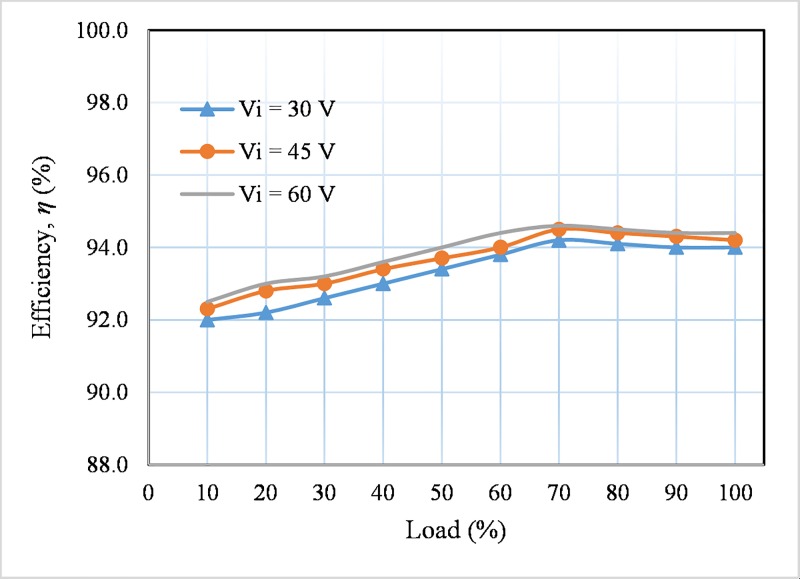
Measured efficiency for different input voltages.

**Fig 14:**
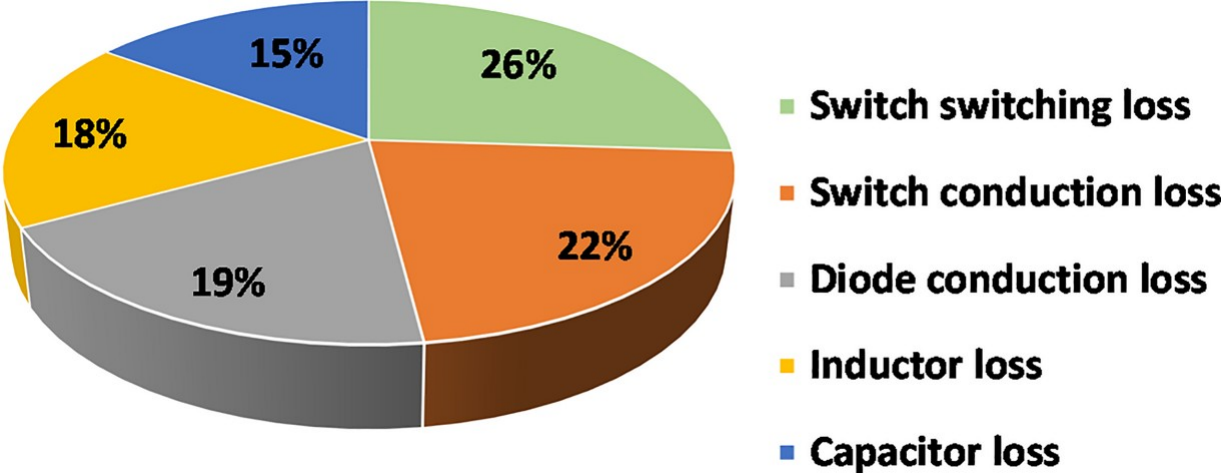
Loss breakdown (calculated value) of the key components of the developed converter.

The Fig 13 presents the efficiency of the developed converter for different input voltages (30, 45 and 60 V) under various load conditions. The efficiencies are measured by measuring the input/output current and voltage by utilizing two current probes with the help of oscilloscope and two Fluke Multimeters. The peak efficiency of the converter is found of 94.6% at 70% load for the input voltage, *V*_*i*_ is 60 V. The voltage output is controlled at 380 V. Fig 14 shows the loss breakdown of the main components of the proposed converter according to the loss distribution analysis described above. From Fig 14, it is seen that the CW capacitors power loss is the lowest of 15%, whereas, the highest power consumption occurs due to the switching losses of 26%.

## Feasibility study

In this section, the feasibility analysis in PV applications of the suggested converter has been described in brief. The feasibility analysis of the developed converter is accomplished for different types of PV panels installed in the Solar Garden at UM Power Energy Dedicated Advanced Center (UMPEDAC), University of Malaya (UM), Kuala Lumpur, Malaysia. Two monocrystalline silicon PV modules (Model: YL275C-30b, *V*_*OC*_ = 39.8 V) of the same power rating (275 W) and maximum power point voltage (*V*_*mpp*_) (31.8 V) have been chosen, one of which is in good physical condition and the other one is partially cracked.

**Fig 15:**
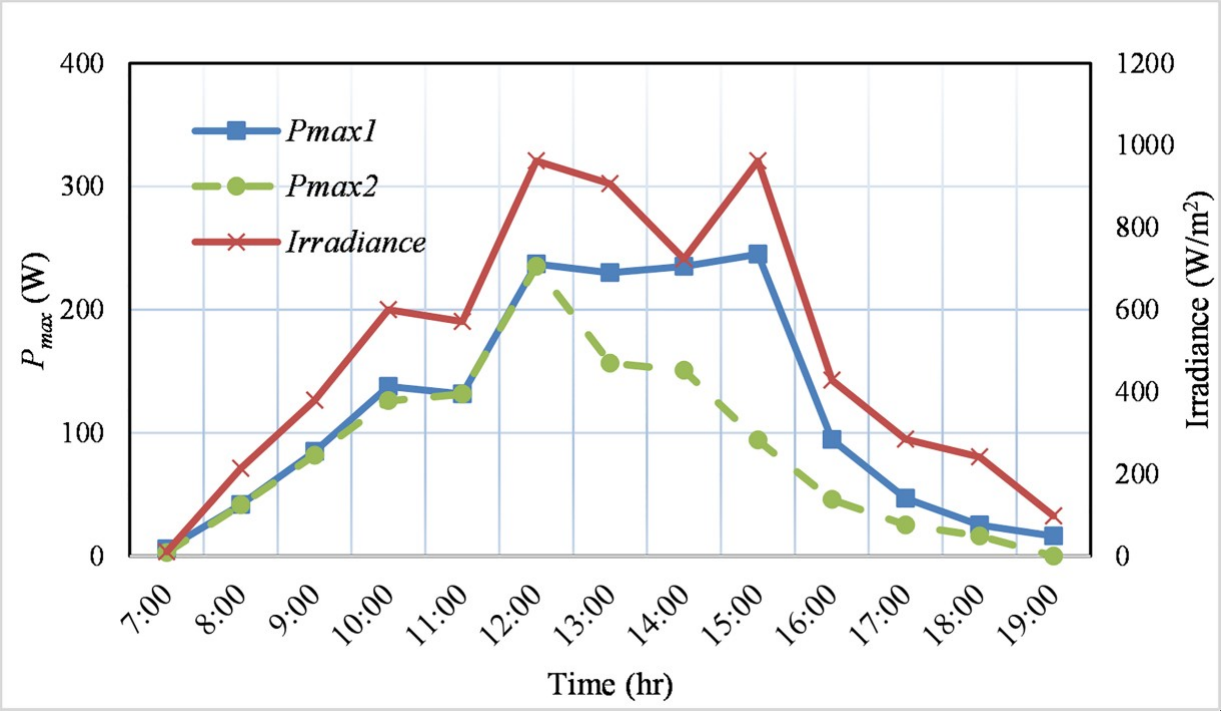
Maximum power of the two monocrystalline type PV panels and irradianceon a sunny day.

**Fig 16:**
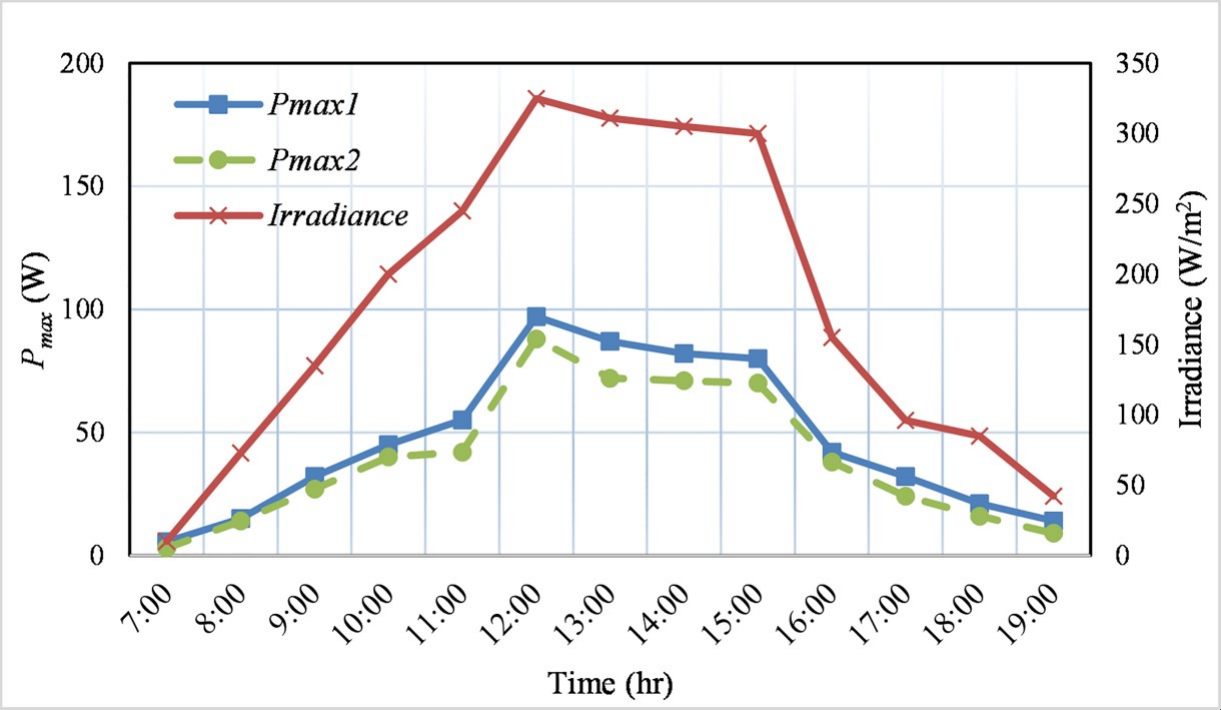
Maximum power of the two monocrystalline type PV panels and irradiance in a cloudy (slight rain) day.

The maximum output power, *P*_*max*_ of these two PV modules and irradiance are recorded in a sunny day (14 October 2017) and a cloudy day (13 October 2017) from 7.00 am to 19.00 pm. Fig 15 illustrates the *P*_*max*_ of the selected PV modules and irradiance in a sunny day. The dashed line represents the power output *P*_*max1*_ of the physically good PV module, while the dotted line displays the partially cracked module power, *P*_*max2*_. The continuous line in Fig 15 presents the solar irradiance. Fig 15 demonstrates that the PV output power fluctuates with the variation of irradiance level and for most of the time during the day both of the module’s output power are higher than 20% of the rated power. On the other hand, Fig 16 presents the output power and irradiance on a cloudy day. In the cloudyday, the irradiance is quite lower compared to that of the sunny day. Therefore, the measured output power is also low as presented in Fig 16, and which is higher than 10% for most of the daytime for both of the modules. Although the extracted power from the PV modules is comparatively low, the proposed converter is well capable (refer to efficiency curve in Fig 13) for these environmental conditions. In addition, from the aforementioned discussion, it is observed that the voltage gain of the developed converter is high enough and it can operate with a wide range of dc input voltage (30 ~ 60 V). Therefore, the above mentioned PV modules with a *V*_*mpp*_ of 31.8 V are suitable for this topology. In case of two strings of PV modules in parallel and each string consists of two modules in series will also be compatible for the developed converter. In addition, other types of PV panels such as thin film, polycrystalline, etc. are also compatible with the designed converter. For example, two thin film panels rated as 135 W, *V*_*mp*_ = 47 V (NS-F135G5) can be connected in parallel providing 270 W output power or two polycrystalline panels rated as 125 W, 17.3 V (PV-AE125MF5N) [36] can also be connected in series to the proposed converter to deliver 250 W and 34.6 V *V*_*mp*_.

## Conclusion

High step-up dc-dc converters are broadly considered as the significant part of the most of the renewable energy systems and many other applications. In this paper, an FB cascaded dc-dc converter has been proposed to attain a high voltage gain with high efficiency. Its circuit operating principle, steady-state analysis, design, and control technique has been explained details. Analysis of voltage stress on different devices has also been carried out and compared with other topologies. Results show that the proposed converter offers lower voltage stress and it does not vary with the number of CW stage changes. Moreover, it requires reduced boost inductance and CW capacitance, which ensure the compactness and lower cost. In addition, the number of major components used in this model are comparatively less than the previous models for the similar voltage step-up ratio. The validation of the theoretical analysis of the suggested converter has been achieved by implementing a hardware prototype. The experimental outcomes agree well with that of the simulations. The efficiency of the converter is found about 94.6% with a peak voltage gain of 11.9. Furthermore, the proposed converter offers lower ripples in input current and output voltage. Finally, a feasibility analysis has been shown with the real PV and environmental data, which ensures the compatibility of the designed converter for a wide-range of PV panels. The designed converter can also adopt efficient Maximum Power Point Tracking (MPPT) technique by introducing of current sensor and slight modification in control algorithm.
